# Identification and characterization of CLEC11A and its derived immune signature in gastric cancer

**DOI:** 10.3389/fimmu.2024.1324959

**Published:** 2024-01-29

**Authors:** Qing Zheng, Zhenqi Gong, Baizhi Li, Runzi Cheng, Weican Luo, Cong Huang, Huaiming Wang

**Affiliations:** ^1^ Department of Gastrointestinal Surgery, The First Affiliated Hospital of Shantou University Medical College, Shantou, China; ^2^ Shantou University Medical College, Shantou, China; ^3^ Department of Ultrasound, The First Affiliated Hospital of Shantou University Medical College, Shantou, China

**Keywords:** Clec11a, gastric cancer, immunotherapy, prognosis, tumor microenvironment

## Abstract

**Introduction:**

C-type lectin domain family 11 member A (CLEC11A) was characterized as a growth factor that mainly regulates hematopoietic function and differentiation of bone cells. However, the involvement of CLEC11A in gastric cancer (GC) is not well understood.

**Methods:**

Transcriptomic data and clinical information pertaining to GC were obtained and analyzed from publicly available databases. The relationships between CLEC11A and prognoses, genetic alterations, tumor microenvironment (TME), and therapeutic responses in GC patients were analyzed by bioinformatics methods. A CLEC11A-derived immune signature was developed and validated, and its mutational landscapes, immunological characteristics as well as drug sensitivities were explored. A nomogram was established by combining CLEC11A-derived immune signature and clinical factors. The expression and carcinogenic effects of CLEC11A in GC were verified by qRT−PCR, cell migration, invasion, cell cycle analysis, and in vivo model analysis. Myeloid-derived suppressor cells (MDSCs), regulatory T cells (Tregs), M2 macrophages, and T cells in tumor samples extracted from mice were analyzed utilizing flow cytometry analysis.

**Results:**

CLEC11A was over-expressed in GC, and the elevated CLEC11A expression indicated an unfavorable prognosis in GC patients. CLEC11A was involved in genomic alterations and associated with the TME in GC. Moreover, elevated CLEC11A was found to reduce the benefit of immunotherapy according to immunophenoscore (IPS) and the tumor immune dysfunction, exclusion (TIDE). After validation, the CLEC11A-derived immune signature demonstrated a consistent ability to predict the survival outcomes in GC patients. A nomogram that quantifies survival probability was constructed to improve the accuracy of prognosis prediction in GC patients. Using shRNA to suppress the expression of CLEC11A led to significant inhibitions of cell cycle progression, migration, and invasion, as well as a marked reduction of *in vivo* tumor growth. Moreover, the flow cytometry assay showed that the knock-down of CLEC11A increased the infiltration of cytotoxic CD8+ T cells and helper CD4+ T into tumors while decreasing the percentage of M2 macrophages, MDSCs, and Tregs.

**Conclusion:**

Collectively, our findings revealed that CLEC11A could be a prognostic and immunological biomarker in GC, and CLEC11A-derived immune signature might serve as a new option for clinicians to predict outcomes and formulate personalized treatment plans for GC patients.

## 1 Introduction

Gastric cancer (GC) remains a considerable threat to human health, ranking the fifth diagnosed cancer and the fourth cause of cancer death in 2020 (1). Due to the delayed diagnosis and absence of efficient treatments, individuals afflicted with advanced GC experience unfavorable prognoses and a short lifespan of merely one year (2). At present, chemotherapy and targeted therapy are the commonly used therapies for advanced GC. However, the outcome of GC patients has not improved substantially due to the toxicity of chemotherapeutic agents, the difficulty of identifying the beneficiary population for targeted therapy agents and drug resistance (3–5).

As one of the most successful immunotherapies, immune checkpoint blockade (ICB) has received approval for the therapy of advanced GC. ICB could amplify endogenous anti-tumor immunity by inhibiting negative regulatory molecules located on the surface of T cells (6). However, the response rate of ICB in GC is not ideal (7–10). A rational explanation for the low response rate is the complex immune suppression mechanisms in the tumor microenvironment (TME) (11). Specifically, various populations of immunosuppressive cells in the TME, including M2 macrophages, myeloid-derived suppressor cells (MDSCs), and regulatory T cells (Tregs), prevent cytotoxic T cells from attacking the tumor to facilitate tumor evasion (12). Encouragingly, targeting some biomarkers, such as growth factors, can transform the inherently immunosuppressive TME into an immunosupportive one (13–16). As a growth factor, the C-type lectin domain family 11 member A (CLEC11A) plays important roles in regulating hematopoietic differentiation and homeostasis, safeguarding against lipotoxicity and severe malaria anemia, and maintaining bone homeostasis (17–25). In cancer research, the prognostic and therapeutic value of CLEC11A has surfaced. CLEC11A was found to be over-expressed and promoted angiogenesis in lung cancer (26). Additionally, the up-regulation of CLEC11A expression in acute myeloid leukemia is associated with a favorable prognosis (27). CLEC11A has also been discovered to regulate the pathogenesis and progression of multiple myeloma (28). Moreover, CLEC11A has been considered a potential deoxyribonucleic acid (DNA) methylation marker for hepatocellular carcinoma and pancreatic cancer (29, 30). Despite the proven role of CLEC11A in the progression of various cancers, the underlying mechanism of CLEC11A remains unclear in GC, especially regarding its role and function in tumor immunity.

**Figure 1:**
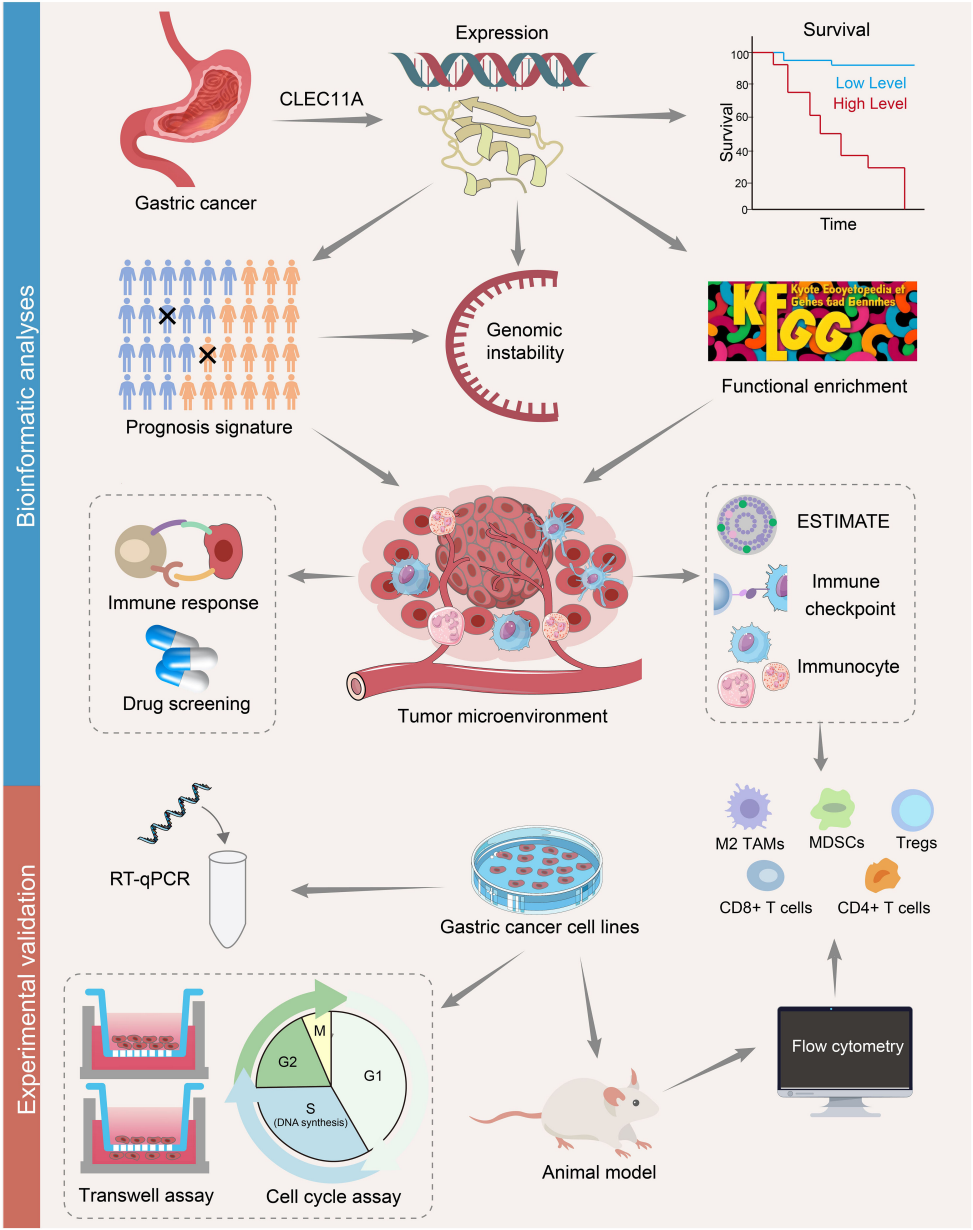
A flowchart of the study design. The mRNA and protein expressions of CLEC11A in GC were investigated using TCGA, GEO, and HPA databases. Kaplan-Meier curves were used to assess the overall survival in CLEC11A subgroups. CLEC11A was involved in genomic instability. The functional enrichment analyses identified CLEC11A’s relevance with cancer immunity. The associations between CLEC11A and ESTIMATE, immune checkpoints, and immunocyte infiltration were further explored. A 6-gene CLEC11A-derived immune signature was constructed to predict prognosis and immune therapy response, and guide precision therapy. The mRNA expression of CLEC11A in GC was verified by qRT−PCR, and the carcinogenic effects of CLEC11A were examined by cell migration, invasion, cell cycle analysis, and in vivo analysis. The associations between CLEC11A and immunocyte infiltration were analyzed utilizing flow cytometry.

RNA sequencing (RNA-seq) has emerged as an omnipresent tool in molecular biology, significantly influencing our comprehension of genomic function. RNA-seq is mainly applied to analyze differential gene expression (DGE) (31). Besides, tumor RNA-seq data can be used to evaluate immune infiltration levels by employing a group of immune-specific marker genes, which may help uncover novel targets for immunotherapy (32–34). Herein, we used bulk tumor RNA-seq information to explore the expression, prognosis value, and genomic alterations of CLEC11A, as well as its immune infiltration in GC. Based on CLEC11A-relevant immune genes, we developed a prognostic signature and evaluated its role in TME. Through laboratory work, we confirmed the increased CLEC11A expression in GC and assessed the impact of CLEC11A on cell cycle, migration, invasion, immunocytes, and *in vivo* tumorigenesis. Our research presented a comprehensive perspective of CLEC11A and introduced a potential selection for predicting the clinical outcomes of GC patients (**Figure 1**).

## 2 Materials and methods

### 2.1 Data collection and processing

RNA-seq information for The Cancer Genome Atlas (TCGA) pan-cancer was downloaded through the Genomic Data Commons data portal. GSE13861, GSE13911, GSE26899, GSE29272, GSE54129, GSE66229, GSE26901, GSE15459, GSE26253, GSE62254, GSE84426, GSE84433, and GSE84437 were obtained from the Gene Expression Omnibus (GEO) database.

### 2.2 Expression analysis

CLEC11A mRNA expression data was assessed from TCGA database, and validated using six datasets obtained from GEO, including GSE13861, GSE13911, GSE26899, GSE29272, GSE54129, and GSE66229. The difference in CLEC11A mRNA expression between tumor and normal samples was calculated using the ‘ggplot2’ package in R (35). The protein expression patterns of CLEC11A were investigated according to the HPA database (36).

### 2.3 Survival analysis

Univariate Cox proportional hazards regression was used to obtain the impact of CLEC11A on overall survival in TCGA cancer types (37). R packages (‘survminer’ and ‘survival’) were used to assess the overall survival rate between CLEC11A subgroups in TCGA-STAD, GSE26899, GSE13861, GSE26901, GSE15459, GSE29272, GSE26253, GSE62254, GSE84426, GSE84433, and GSE84437, respectively (38).

### 2.4 Analysis of genomic alteration and epigenetic modification

The frequency of two types of genomic alterations, specifically mutations and amplifications, was analyzed employing the “Cancer Types Summary” module of the web-based tool cBioPortal (https://www.cbioportal.org/) (39). Tumor mutation burden (TMB) was computed by the R software package “Maftools” (40). Homologous recombination deficiency (HRD), loss of heterozygosity (LOH), ploidy, and microsatellite instability (MSI) were obtained from previous research (41). The associations between CLEC11A mRNA expression and TMB, MSI, ploidy, HRD, LOH were explored and visualized by ‘ggplot2’ package in R (35). The correlation between CLEC11A expression and 4 DNA methyltransferase genes (42), 5 mismatch repair (MMR) genes (43), and 44 RNA modification genes (44–46) was visualized by R package ‘ComplexHeatmap’ (47). The gene mutation frequency and chromosomal gain/loss were analyzed between CLEC11A subgroups.

### 2.5 Functional analysis

Based on the median CLEC11A mRNA expression, the TCGA-STAD samples were categorized into two groups. With |log(fold change)| > 1 and adjusted p-value < 0.01, the differentially expressed genes were determined and included in the Kyoto Encyclopedia of Genes and Genomes (KEGG) and gene ontology (GO) analyses by the R software package “ClusterProfiler” (48). The molecular mechanisms and immune landscapes based on specific groups of genes were determined by Gene Set Enrichment Analysis (GSEA) (49).

### 2.6 TME analysis

Infiltration of immune and stromal cells was evaluated in GC by the ESTIMATE (Estimation of STromal and Immune cells in MAlignant Tumours using Expression data) algorithm (50). Immune cells and immunomodulators associated with CLEC11A in GC were obtained from TISIDB, an online database that provides interaction information between tumors and the immune system (51). In accordance with mRNA expression data, CIBERSORT algorithm was conducted to characterize the TME cell composition in GC tissues (52). Single-sample GSEA (ssGSEA) analysis was conducted for quantitatively elucidating immune function enrichment values (53).

### 2.7 Immunotherapy response

The expression patterns of CLEC11A in GC molecular subtypes were assessed from TISIDB (51, 54). Tumor immune dysfunction, exclusion (TIDE), immunophenoscore (IPS), and several biomarkers (TMB, microsatellite stable (MSS), MSI) were evaluated to predict ICB responses (55, 56).

### 2.8 Development of a CLEC11A-derived immune signature

Immunoinhibitors and immunostimulators correlated with CLEC11A (Spearman correlation test, P less than 0.05) from TISIDB were selected for univariate Cox regression. After that, significant prognostic genes were included in the random forest survival analysis (57). With the optimal cutoff, the GC samples were partitioned into two groups. The relationships between the risk score and clinical outcomes were examined through Kaplan-Meier analyses. The independence of CLEC11A-derived immune signature in prognosis prediction was verified by univariate and multivariate Cox regression analyses. ROC curves were employed further to validate the efficacy and precision of risk scores in predicting one-, three-, and five-year outcomes.

### 2.9 Nomogram establishment

The clinical factors that have independent prognostic value were combined with the CLEC11A-derived immune signature to construct a nomogram. Subsequently, the independence of the nomogram was determined in univariate and multivariate Cox regressions. The consistency between the actual and observed survival rates of the nomogram was evaluated using calibration curves.

### 2.10 Genomic variation landscape

The Mutation Annotation Format (MAF) file from TCGA, containing somatic variants, was analyzed using the R package “maftools” (40). Copy number variation (CNV) data was obtained from UCSC Xena, an online platform for accessing genomic datasets (https://xenabrowser.net/datapages/) (58). To annotate the genes in the CNV region, the genome research consortium Human Build 38 was used.

### 2.11 Drug sensitivity

The semi-inhibitory concentration (IC50) values of drugs were determined by the R package “pRRophetic,” which allows the prediction of drug response based on pharmacogenomic data (59).

### 2.12 Cell culture

The normal human gastric mucosa cells (GES-1) and human GC cells (BGC-803, AGS, HGC-27, SGC-7901, and BGC-823) were obtained from ATCC (Shanghai, China) and cultured in McCoy’s 5a Medium (Gibco, Grand Island, NY, USA), supplemented with 10% fetal bovine serum (Gibco, sourced from Australia) and 1% streptomycin/penicillin, at a temperature of 37°C with 5% CO_2_.

### 2.13 RNA interference

GC cells with reduced expression of CLEC11A were produced using 5 mg/ml polybrene and lentiviruses (multiplicity of infection [MOI], 100; packaged by Cyagen Biosciences). Stable CLEC11A-downregulated cells (sh-CLEC11A cells) were screened using Puromycin, and the control shRNA (sh-control) was obtained from Cyagen Biosciences. Cell transfection was performed in line with the manufacturer’s instructions. The sequences of shRNA utilized were as follows: sh-CLEC11A: 5’-TGAGGACATCGTCACTTACATCTCGAGATGTAAGTGACGATGTCCTCA-3’; sh-Control: 5’-CCTAAGGTTAAGTCGCCCTCGCTCGAGCGAGGGCGACTTAACCTTAGG-3’.

### 2.14 RT-qPCR analysis

Total RNA was extracted from the cells using TRIzol reagent (Invitrogen, USA). Subsequently, reverse transcription was performed using the PrimeScript RT Reagent Kit acquired from TaKaRa. The following PCR conditions were employed on the StepOnePlus PCR System (TaKaRa) using 2x RealStar Power SYBR Mixture (TaKaRa): an initial predenaturation at 95°C for 2 min, then 95°C for 15 s, 60°C for 30 s, and 72°C for 30 s, for a total of 40 cycles. The PCR amplification primer sequences were as follows:: CLEC11A, forward: 5’-CTGCCGGAACTGTTGAGGG-3’, and reverse: 5’-CCCAGGATGTAAGTGACGATGT-3’; β-actin, forward: 5′ -TCCATCATGAAGTGTGACGT-3′, reverse: 5′ GAGCAATGATCTTGATCTTCAT-3′. The relative RNA expression was determined using the comparative Ct method and normalized to β-actin transcripts. Each assay was repeated at least three times.

### 2.15 Transwell assay

A total of 1 × 10^5^ cells were seeded onto a fibronectin-coated polycarbonate membrane insert in a transwell apparatus manufactured by Corning (NY, USA), with a pore size of 0.8 mm. In the lower chamber of the transwell, 600 μl of RPMI 1640 medium supplemented with fetal bovine serum from Beyotime Institute of Biotechnology was added as a chemoattractant. Following a 12-hour incubation, the insert was carefully washed with PBS to remove any non-adherent cells from the upper surface. Next, the GC cells that migrated through the membrane and attached to the lower surface of the insert were fixed using 4% formaldehyde. To visualize and quantify the migrated cells, the fixed cells were stained with a 0.2% crystal violet solution obtained from Shanghai Qiaoxing Trading Corporation in Shanghai, China. Cell counts were determined using ImageJ software, and photographs were captured. The Matrigel invasion assay was conducted following a procedure similar to the cell migration assay described above. However, in the Matrigel invasion assay, the transwell membrane was pre-coated with ECMatrix™, and the cells were incubated for 14 hours. Each experiment was repeated a minimum of three times.

### 2.16 *In vivo* cell proliferation assay

All animal studies were performed in line with the guidelines set by the National Regulation of China for the Care and Use of Laboratory Animals. Animal models were constructed using female BALB/c mice (4~6 weeks old, purchased from the Laboratory Animal Center of Southern Medical University). For each group, 5 × 10^6^ treated MFC cells (sh-CLEC11A and sh-control) were collected and subcutaneously injected into female BALB/c mice (n = 5). After 7 days of subcutaneous tumor formation, the tumor volume of mice was measured every 96 hours by the electronic scale and vernier caliper. After the initiation of treatment for 27 days, all mice were euthanized by cervical dislocation, and the tumor was excised for further experiments.

### 2.17 Cell cycle assay

Following a 72-hour transfection with sh-control and sh-CLEC11A, the cells were washed with PBS and then fixed in 1 mL of pre-cooled 70% ethanol for 4 hours at -20°C. After centrifugation at 200 x g for 5 minutes, the cells were resuspended in 0.5 mL of PBS containing 40 μg/mL of propidium iodide solution and 100 μg/mL of RNase A. Subsequently, the cells were incubated at 37°C for 30 minutes and then analyzed using flow cytometry.

### 2.18 Flow cytometry immunophenotyping analysis

Tumors were harvested from mice in different groups to investigate the immune cells in sh-control and sh-CLEC11A tumors. Single-cell suspensions were prepared via filtration using a 70 μm mesh after digestion with collagenase IV (0.3 mg/mL) for one h at 37°C. Next, the harvested cells were incubated with CD16/CD32 antibodies to block non-specific binding, followed by culture in eBioscience™ Fixable Viability Dye eFluor™ 506. Subsequently, the percentage of multiple immune cells was examined by flow cytometry after staining with several antibodies: CD8+ T cells (CD8, CD3, and CD45 antibodies), CD4+ T cells (CD3, CD45, and CD4 antibodies), MDSCs (CD45, Gr-1, and CD11b antibodies), Tregs (CD45, CD25, CD3, CD4, and Foxp3 antibodies), and macrophages (CD45, CD11b, F4/80, CD206, and CD86 antibodies).

### 2.19 Statistical analysis

Statistical analyses were conducted using GraphPad Prism v9.5 and R software v4.0.3. The statistical significance of the expression differences between different groups was assessed using a non-parametric Wilcoxon rank sum test. The log-rank test was used to evaluate the prognostic significance. The Spearman method was employed for conducting the correlation analysis. Univariate and multivariate Cox regression analyses were used to identify the related factors affecting the overall survival of GC patients. The detailed Cox regression results can be found in **Supplementary Table 1**. Statistical significance was established at a P < 0.05.

## 3 Results

### 3.1 CLEC11A was up-regulated in GC

**Figure 2:**
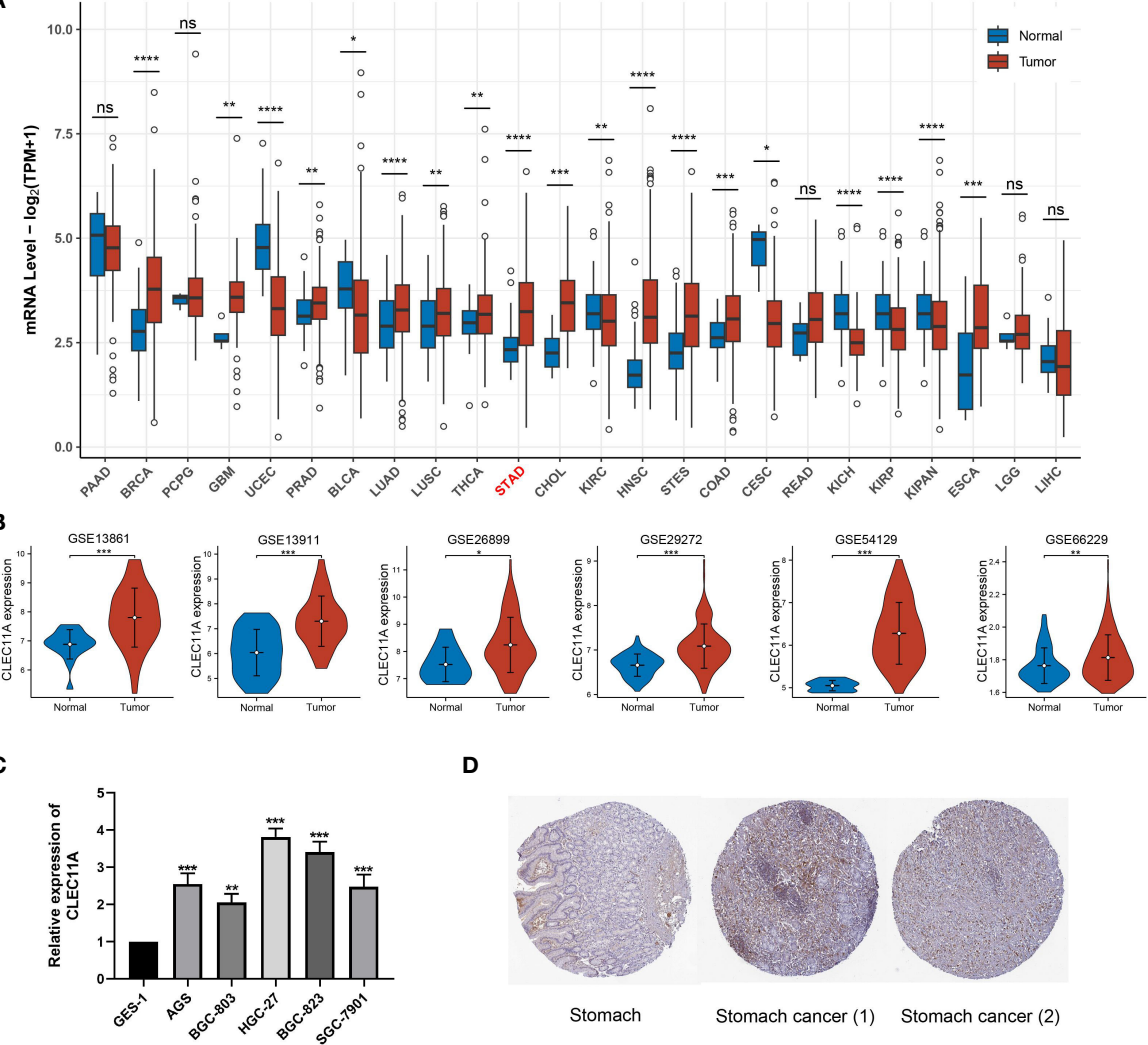
CLEC11A was over-expressed in GC. **(A)** CLEC11A mRNA expression across TCGA pan-cancer. **(B)** CLEC11A mRNA expression in GSE13861, GSE13911, GSE26899, GSE29272, GSE54129, and GSE66229. **(C)** The CLEC11A mRNA expression in GC was verified by PCR analysis. **(D)** Immunohistochemical analysis of CLEC11A in GC and normal stomach tissues by HPA database. * P < 0.05, ** P < 0.01, *** P < 0.001, **** P < 0.0001, ns: not significant.

To identify the CLEC11A expression in pan-cancer, we used TCGA RNA-seq data to determine the differential expression of CLEC11A in tumor tissues compared to normal tissues. Our analysis revealed that CLEC11A exhibited a significant upregulation in various cancers, including GC (**Figure 2A**, p<0.001). The upregulated CLEC11A expression in GC was confirmed by GSE13861, GSE13911, GSE26899, GSE29272, GSE54129, and GSE66229 (**Figure 2B**). Additionally, in TCGA-STAD, the expression of CLEC11A varied among different T staging (Chisq.test, p = 0.021; **Supplementary Table 2**).

Moreover, we used RT-qPCR analysis to assess the CLEC11A mRNA expression levels in GC cells. Our results revealed a significant increase in the relative mRNA expression of CLEC11A in six different human GC cell lines (BGC-803, AGS, SGC-7901, BGC-823, and HGC-27) when compared to GES-1, a human gastric mucosal epithelial cell line (**Figure 2C**). Among the 6 GC cell lines, HGC-27 cells exhibited the most elevated expression levels of CLEC11A, suggesting HGC-27 cells are a viable model for investigating the functions of CLEC11A through a loss-of-function approach.

Using the HPA database, we examined the CLEC11A protein expression in GC. The immunohistochemical images uncovered that the expression of CLEC11A protein was elevated in GC (**Figure 2D**).

### 3.2 CLEC11A was linked to an unfavorable prognosis in GC

**Figure 3:**
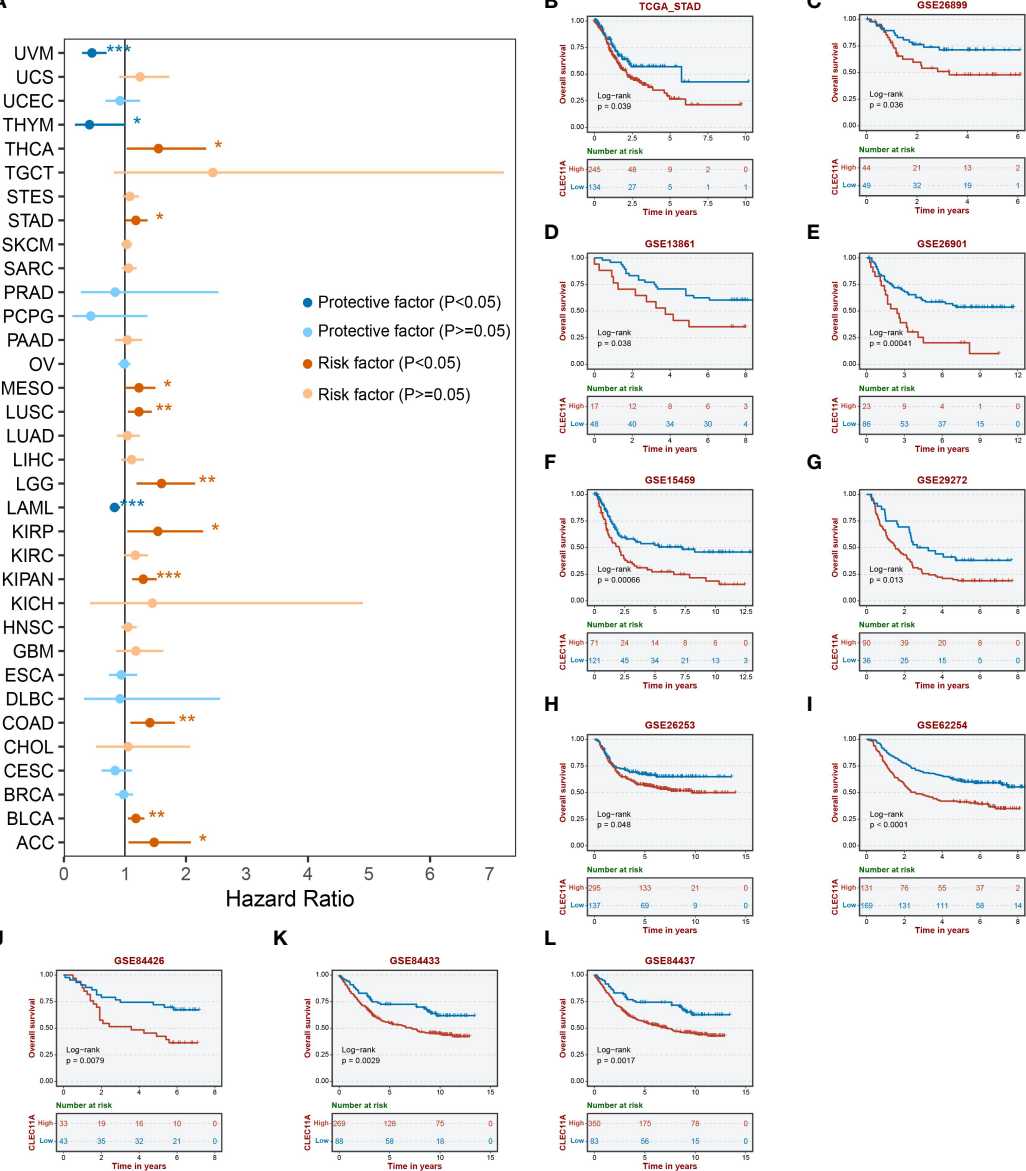
Prognosis analyses of CLEC11A in GC. **(A)** Cox regression of CLEC11A across TCGA cancer types. Overall survival analyses of CLEC11A in **(B)** TCGA-STAD, **(C)** GSE26899, **(D)** GSE13861, **(E)** GSE26901, **(F)** GSE15459, **(G)** GSE29272, **(H)** GSE26253, **(I)** GSE62254, **(J)** GSE84426, **(K)** GSE84433, and **(L)** GSE84437.

To determine the prognostic significance of CLEC11A, we conducted univariate Cox regression to explore the relationship between CLEC11A and patient survival time across TCGA cancers. We observed that high expression levels of CLEC11A could be a negative prognostic factor for GC patients (HR = 1.47, p < 0.05; **Figure 3A**). Additionally, we examined the correlation between CLEC11A expression and overall survival in GC. As shown in **Figures 3B–L**), patients exhibiting high expression of CLEC11A experienced shorter overall survival time compared to those with low expression of CLEC11A in TCGA-STAD (p < 0.05), GSE26899 (p < 0.05), GSE13861 (p < 0.05), GSE26901 (p < 0.001), GSE15459 (p < 0.001), GSE29272 (p < 0.05), GSE26253 (p < 0.05), GSE62254 (p < 0.001), GSE84426 (p < 0.01), GSE84433 (p < 0.001), and GSE84437 (p < 0.01).

### 3.3 CLEC11A was associated with genomic instability and epigenetic modification

**Figure 4:**
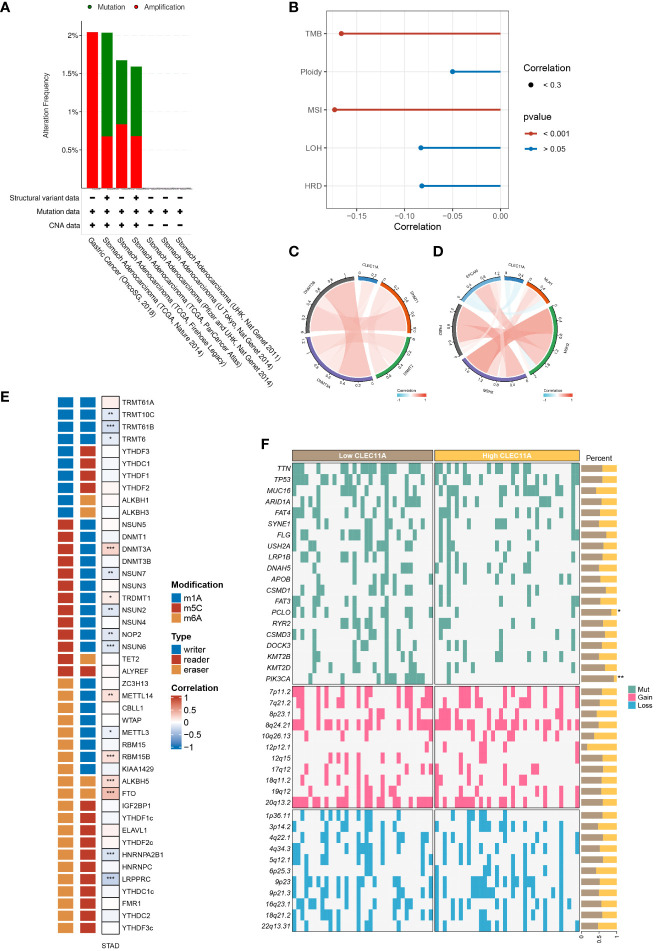
CLEC11A was related to epigenetic modulations and genomic instability in GC. **(A)** The genomic alterations of CLEC11A in GC were explored by the cBioPortal online web tool, including mutation and amplification. **(B)** The correlation between CLEC11A and genomic heterogeneity. The correlation between CLEC11A and **(C)** 4 methyltransferases, **(D)** 5 MMR genes, and **(E)** 44 RNA modulations. **(F)** The gene mutation frequency and chromosomal gain/loss were analyzed between CLEC11A subgroups in TCGA-STAD. * P < 0.05, ** P < 0.01, *** P < 0.001.

Genomic instability is a crucial factor that influences gene expression and the TME, which generally promotes tumor progression (60, 61). To investigate the genomic alterations of CLEC11A, we employed the cBioportal to examine the alteration frequencies of CLEC11A in GC (**Figure 4A**). The results showed that the main types of CLEC11A alteration in GC are mutation and amplification. Furthermore, we calculated the TMB, ploidy, LOH, HRD, and MSI correlations with CLEC11A in GC due to the unignorable impact of these genomic alterations on patient prognosis and therapeutic responses (62–66). The results (**Figure 4B**) uncovered a negative correlation between CLEC11A and TMB (R = -0.166, p < 0.001) as well as MSI (R = -0.173, p < 0.001). No statistically significant correlation was observed between CLEC11A and HRD (p = 0.098), LOH (p = 0.097), ploidy (p = 0.321). MMR genes are responsible for fixing errors that occur during DNA replication, which helps maintain cancer genomic stability (67). Thus, we examined the correlations between CLEC11A and MMR genes (PMS2, MSH6, MSH2, MLH1, and EPCAM). The results revealed a negative correlation between CLEC11A and multiple MMR genes (**Figure 4D**). Furthermore, we explored the relationship between gene mutation frequency, chromosome gain/loss, and CLEC11A expression in patients with GC (**Figure 4F**). Patients with high CLEC11A expression had lower mutation frequencies in PCLO (p < 0.05) and PIK3CA (p < 0.01) than those with low levels of CLEC11A. However, no significant differences in chromosome gain/loss were found between the high- and low-expression groups of CLEC11A.

Epigenetic changes play a vital role in the initiation of carcinogenesis, tumor progression, and metastasis (68, 69). We sought the influences of CLEC11A on cancer epigenetic modulations. As illustrated in **Figure 4C**, among 4 DNA methyltransferases, CLEC11A exhibited a weak negative correlation with DNMT3B in GC (Cor = -0.02, p < 0.001). We further explored the correlation between CLEC11A and RNA modulator genes. There was a significant association between high CLEC11A expression and RNA modulator genes in GC, specifically m6A, m5C, and m1A (**Figure 4E**), suggesting the involvement of CLEC11A in RNA modifications.

### 3.4 GO and KEGG

**Figure 5:**
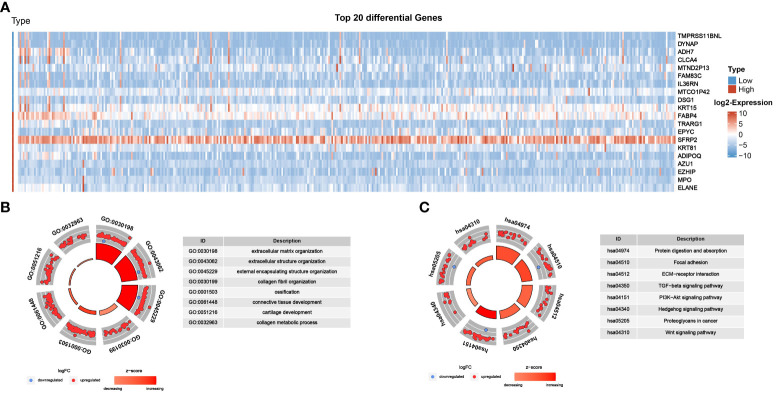
Functional analysis of CLEC11A in GC. **(A)** Top 20 differential genes between different CLEC11A expression subgroups. **(B)** GO analysis and **(C)** KEGG analysis of differential genes of CLEC11A.

To investigate the biological significance of CLEC11A in GC, we captured the co-expressed genes for conducting functional enrichment analysis. As shown in **Figure 5A**, the top 20 differential genes with |log fold change>1| were detected. Using the GO approach, we observed that the biological processes were mainly concentrated in the extracellular matrix (**Figure 5B**). Moreover, the results of KEGG indicated that CLEC11A exhibited a close association with ECM-receptor interaction, TGF-beta signaling pathway, focal adhesion, and protein digestion and absorption (**Figure 5C**).

### 3.5 Reduced CLEC11A inhibited cell cycle progression, migration and invasion

**Figure 6:**
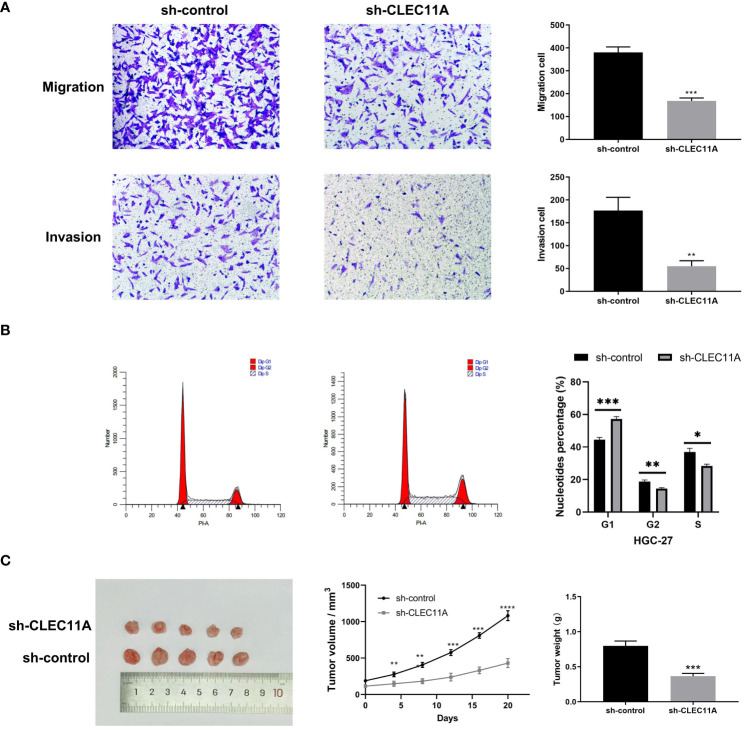
The oncogenic effect of CLEC11A in GC. **(A)** Migration and invasion assays. **(B)** Cell cycle assay. **(C)** Reduced CLEC11A expression inhibited tumor growth in vivo. * P < 0.05, ** P < 0.01, *** P < 0.001.

Given the functional enrichment analyses suggested that CLEC11A may play a role in GC development, we attempted to reduce CLEC11A expression to understand the biological function of CLEC11A in GC. Concretely, we utilized a lentiviral vector with shRNA targeting and suppressing the expression of CLEC11A in HGC-27 cells, a GC cell line with elevated levels of CLEC11A. Cell migration and invasion are vital stages in tumor progression and metastasis. During the investigation into the migratory and invasive capabilities of HGC-27 cells, we found that the downregulation of CLEC11A resulted in a significant decrease in cell migration and invasion compared to the sh-control cells (P < 0.01) (**Figure 6A**). Additionally, we assessed the impact of CLEC11A knock-down on cell cycle progression. Through flow cytometry analysis, we observed a significant decrease in the proportion of cells in the G2/S phase and an increase in the G1 proportion in CLEC11A-deficient cells compared to the sh-control HGC-27 cells (P < 0.05) (**Figure 6B**).

### 3.6 Reduced CLEC11A inhibited tumor growth in vivo

Besides investigating the biological functions of CLEC11A in vitro, we also evaluated its in vivo role using a tumor transplantation model. Following the subcutaneous transplantation of cells containing sh-control or sh-CLEC11A lentiviral vectors into nude mice, we observed and recorded the progression of tumor growth for a duration of 20 days. **Figure 6C** demonstrated a significant inhibition of tumor growth in mice through CLEC11A knockdown. Analysis of tumor volume and weights revealed that shRNA-CLEC11A cells yielded markedly smaller tumors compared to sh-control cells (P < 0.001) (**Figure 6C**).

### 3.7 CLEC11A mediated immune infiltration in TME

**Figure 7:**
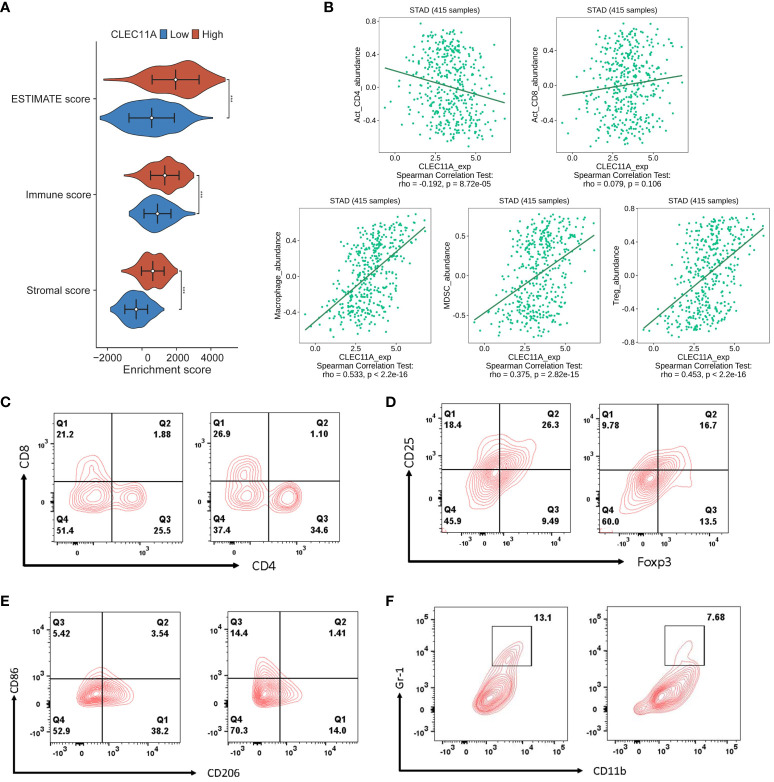
CLEC11A mediated immune lymphocytes in TME. **(A)** Distributions of TME scores between CLEC11A subgroups. **(B)** Correlations between CLEC11A and immune lymphocytes in TISIDB. Flow cytometry immunophenotyping analysis of the populations of **(C)** cytotoxic CD8+ and helper CD4+ T cells, **(D)** Tregs, **(E)** M2 macrophages, and **(F)** MDSCs in MFC tumor-bearing mice after reducing CLEC11A expression.

**Figure 8:**
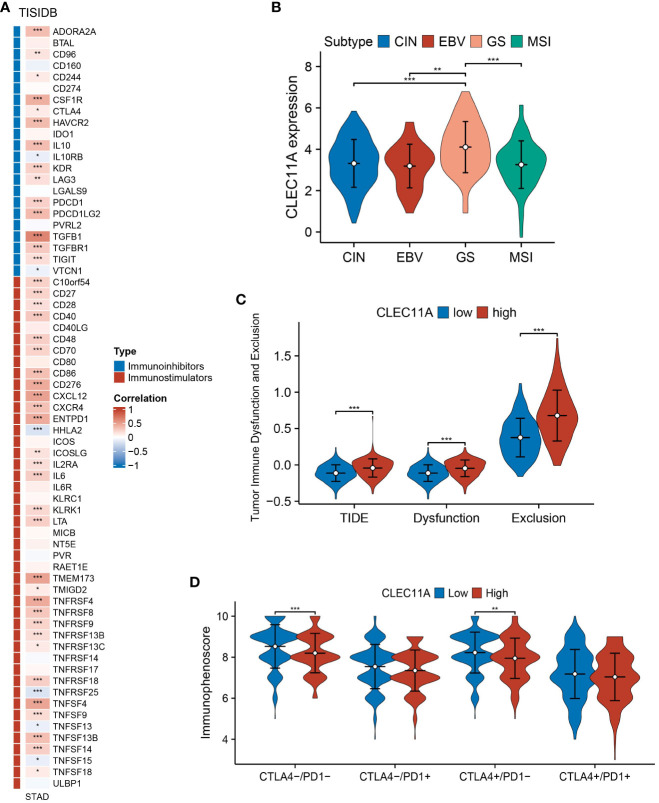
Immune-related genes and immune response analyses. **(A)** Expression correlations between CLEC11A and immunoinhibitors, immunostimulators according to TISIDB database. **(B)** The expression patterns of CLEC11A in four TCGA molecular subtypes of GC. **(C)** TIDE and **(D)** IPS between CLEC11A subgroups. * P < 0.05, ** P < 0.01, *** P < 0.001.

It is well known that TME plays a critical role in regulating malignancy progression and modulating therapeutic response (70). A better understanding of the TME could contribute to the evolution of immunotherapy for GC (71). To investigate whether CLEC11A was connected with TME, we used the ESTIMATE algorithm to compute the stromal score, immune score, and ESTIMATE score in the TCGA-STAD cohort. As shown in **Figure 7A**, the ESTIMATE score (p <0.001), stromal score (p <0.001), and immune score (p <0.001) were significantly higher in the high CLEC11A expression group. Next, we performed the correlation analysis between CLEC11A expression and immune cells in the TISDB (**Figure 7B**). Our findings revealed that CLEC11A was correlated with active CD4+ T cells (Spearman: R = -0.192, p < 0.001), macrophages (Spearman: R = 0.533, p < 0.001), MDSCs (Spearman: R = 0.375, p < 0.001), and Tregs (Spearman: R = 0.453, p < 0.001) infiltration. Using flow cytometry analysis, we explored the impact of decreased CLEC11A expression on immune cells. As shown in **Figure 7C**, by knock-down of CLEC11A expression, both cytotoxic CD8+ and helper CD4+ T cells infiltrated into the tumors effectively. Moreover, knocking down endogenous CLEC11A could dramatically decrease the percentage of Tregs, M2 macrophages, and MDSCs (**Figures 7D–F**). The abundance of immune cells in the TME significantly correlates with the survival prognosis of cancer patients (72, 73). In TCGA-STAD, we found that high levels of M2 macrophages and T cells CD4 memory resting were associated with poor prognosis in patients, while high levels of T cells CD8 and T cells CD4 memory activated indicate a favorable prognosis (**Supplementary Figure 1**). We also detected that the CLEC11A was correlated with multiple immunoinhibitors and immunostimulators in TISIDB (**Figure 8A**).

### 3.8 Increased expression of CLEC11A was associated with a diminished response to immunotherapy

In 2014, a study based on the TCGA program proposed four molecular subtypes of GC: chromosomal instability (CIN), microsatellite instability (MSI), Epstein–Barr virus (EBV), and genomically stable (54). Specific subtypes within GC, such as MSI-H tumors, demonstrate high sensitivity to immunotherapy (74). When compared to the MSI subtype, we found that CLEC11A expression was higher in genomically stable gastric tumors (**Figure 8B**); a molecular subtype indicates poor prognosis and few clear treatment targets (54, 75).

The TIDE is an algorithm designed to predict the effectiveness of tumor immunotherapy, which considers both Dysfunction and Exclusion in the TME (55). Based on the TIDE analysis, we found that patients with high CLEC11A expression had significantly higher TIDE scores, dysfunction scores and exclusion scores compared to those with low CLEC11A expression (**Figure 8C**).

The IPS has been demonstrated as a dependable predictor of immune checkpoint inhibitors (ICIs) treatment (56). We calculated the IPS of GC patients from TCGA-STAD and found that patients with low levels of CLEC11A expression presented significant therapeutic benefits from ICI treatments (CTLA4-/PD-1- and CTLA4+/PD-1-) (**Figure 8D**).

### 3.9 The CLEC11A-derived immune signature for GC prognosis

**Figure 9:**
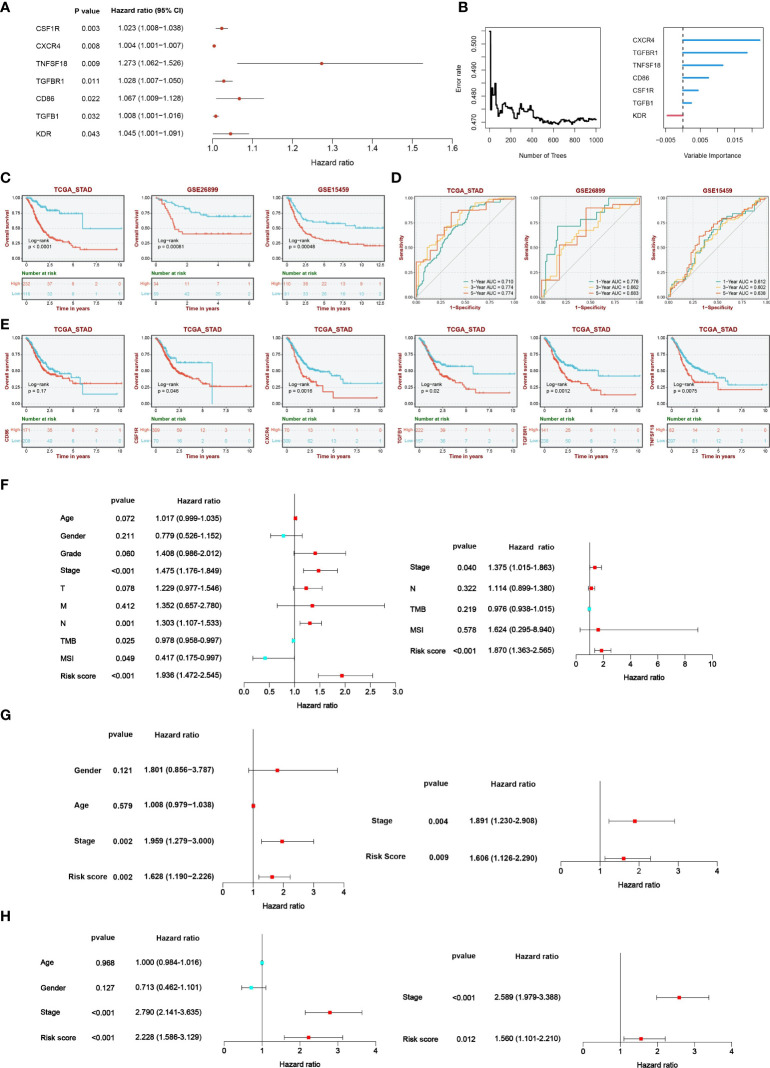
A CLEC11A-derived immune signature was developed and validated. **(A)** The results of univariate Cox regression were presented by forest plot. **(B)** The importance of CLEC11A-related immune genes was calculated by random survival forest analysis. **(C)** Overall survival analysis between risk score subgroups in TCGA-STAD cohort, GSE26899, and GSE15459. **(D)** ROC curves of CLEC11A-derived immune signature in predicting one, three, and five-year overall survival in the TCGA training set, GSE26899 and GSE15459. **(E)** Five genes (CSF1R, CXCR4, TGFB1, TGFBR1, and TNFSF18) included in the signature showed associations with the overall survival of patients in the TCGA-STAD cohort. Independent prognostic analyses of the clinical features and CLEC11A-derived immune signature in **(F)** TCGA-STAD cohort, **(G)** GSE26899, and **(H)** GSE15459.

According to TISIDB, we detected 33 immunostimulators and 16 immunoinhibitors that were significantly associated with CLEC11A. To examine the prognostic values of CLEC11A-associated immunomodulators in GC, we conducted a univariate Cox regression analysis on these variables. This analysis revealed that seven genes exhibited a p-value less than 0.05, indicating potential significance in predicting prognosis (**Figure 9A**). The importance of the prognostic genes mentioned above was ranked using random survival forest analysis (**Figure 9B**). Based on the importance score being greater than zero, a total of six immune genes were identified: CSF1R, TGFB1, TGFBR1, CD86, CXCR4, and TNFSF18 (**Figure 9B**). Using the Kaplan-Meier curves, we found that patients with low-risk scores had significantly longer survival compared to patients with high-risk scores (log-rank test, P<0.001) (**Figure 9C**). Moreover, we found that five genes (CSF1R, CXCR4, TGFB1, TGFBR1, and TNFSF18) included in the signature exhibited significant associations between the prognosis of GC (log-rank test, p<0.05) (**Figure 9E**). When testing one-, three-, and five-year overall survival probabilities, we found that CLEC11A-derived immune signature possessed good potency in the TCGA training cohort and external validation cohorts (GSE26899 and GSE15459) (**Figure 9D**). By conducting univariate and multivariate Cox regressions, the CLEC11A-derived immune signature was considered an independent prognostic indicator of GC (**Figures 9F–H**).

### 3.10 Development and verification of a nomogram

**Figure 10:**
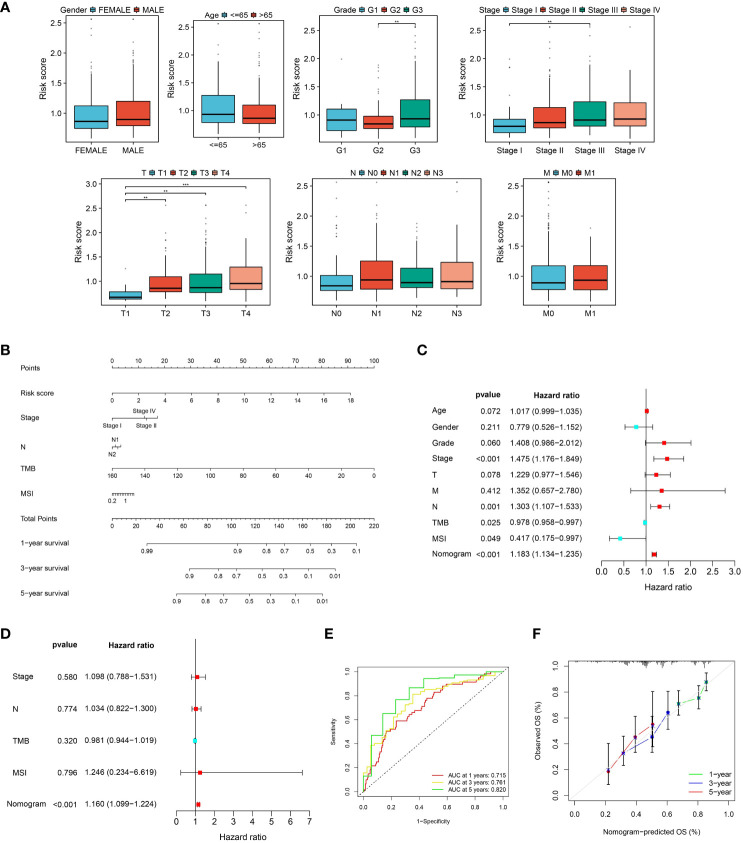
A nomogram was developed and validated. **(A)** The distributions of risk scores in clinical features. **(B)** Nomogram construction based on the CLEC11A-derived immune signature and clinical characteristics, including stage, N, TMB, and MSI. **(C, D)** Independent prognostic analyses of the CLEC11A-derived immune signature and clinical features in the TCGA-STAD cohort. **(E)** ROC curves showed the prediction performances of the nomogram in one-, three-, and five-year overall survival. **(F)** Calibration curves of the nomogram for one, three, and five-year overall survival.

Since clinical features are typically used in clinical practice to evaluate the survival outcome of GC patients, we examined the associations between the CLEC11A-derived immune signature and multiple clinical features. Within the TCGA-STAD cohort, we found that the distribution of risk scores was significantly different in grade, stage, and T (p < 0.01, Dunn’s test) (**Figure 10A**). With the aim of making the CLEC11A-derived immune signature more clinically applicable, we combined the prognostic signature and independent clinical features to establish a nomogram (**Figure 10B**). To evaluate the independent prognostic value of the nomogram in GC, we performed univariate and multivariate Cox regression analyses on overall survival using the TCGA-STAD dataset. The results demonstrated that the nomogram significantly influenced the overall survival rate in the univariate analysis (HR > 1, p < 0.001) (**Figure 10C**). Furthermore, the nomogram showed consistent value as an independent prognostic factor for overall survival in the multivariate analysis (HR = 1.160, 95% CI 1.099-1.224, p < 0.001) (**Figure 10D**). The area under the curve (AUC) of the nomogram reached 0.715, 0.761, and 0.820 at one-, three-, and five-year intervals, respectively, signifying a robust level of predictive accuracy (**Figure 10E**). The calibration curves demonstrated a good fit between the predictions of the nomogram and the actual observations (**Figure 10F**). These findings indicated that the CLEC11A-based nomogram is a dependable and accurate tool for predicting prognosis in GC.

### 3.11 Genomic variation landscape of CLEC11A-derived immune signature

**Figure 11:**
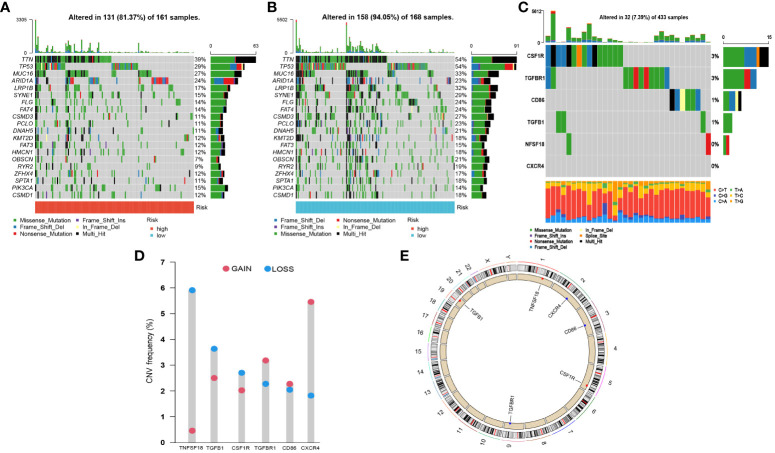
Somatic mutations and CNVs analysis in GC patients. **(A, B)** Waterfall graphs illustrated the mutational landscape in risk score subgroups. **(C)** Genetic alteration of 6 signature genes. **(D)** CNV frequencies of 6 signature genes. **(E)** Genomic positions of 6 signature genes. The bands on the inner circle represented the corresponding expression levels.

To determine the genomic variation of CLEC11A-derived immune signature, we utilized the waterfall graphs to visualize the mutational landscape of the top 20 genes occurring in the risk score subgroups (**Figures 11A, B**). GC patients in the high-risk group were discovered to have fewer TNN and TP53 mutations than GC patients in the low-risk group. When summmaring the occurrence of CNVs and somatic mutations of 6 signature genes, we observed that these genes were mutated in 32 GC patients with a frequency of 7.38% (**Figure 11C**). Moreover, we found that the frequencies of copy number gain and loss were common in 6 signature genes (**Figure 11D**). The positions of signature genes on chromosomes were visualized in **Figure 11E**.

### 3.12 The TME characteristics of CLEC11A-derived immune signature

**Figure 12:**
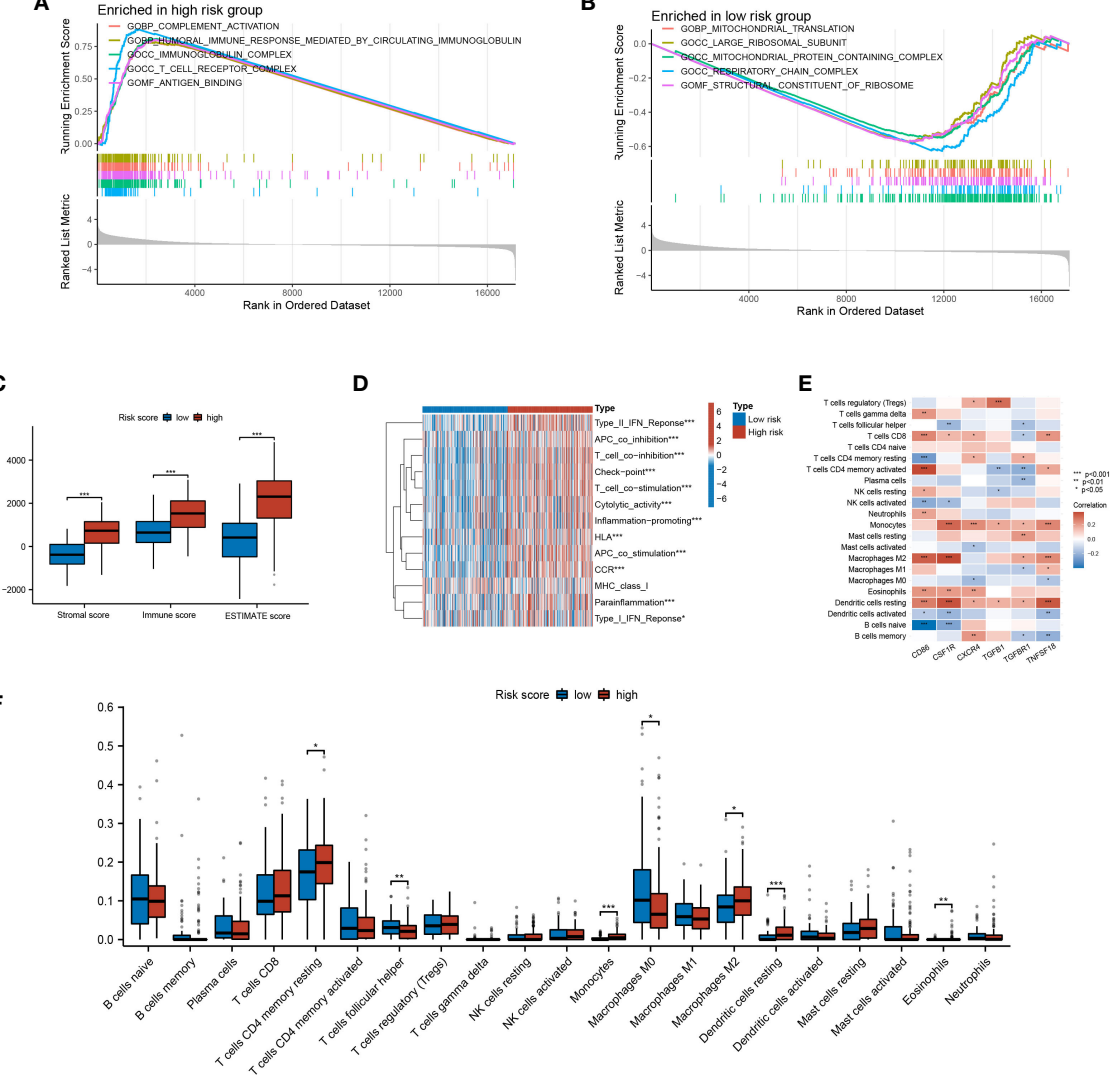
The TME characteristics of CLEC11A-derived immune signature. **(A, B)** GO terms enriched in risk score subgroups were determined by GSEA analysis. **(C)** Immune fractions between the risk score subgroups were quantified by TME scores. **(D)** Differences of immune-related pathways between risk score subgroups. **(E)** The relationships between TME infiltrated cells and genes included in CLEC11A-derived immune signature. **(F)** CIBERSORT algorithm quantified the TME infiltrated cells between risk score subgroups. * P < 0.05, ** P < 0.01, *** P < 0.001.

To understand the molecular mechanisms mediated by CLEC11A-derived immune signature in GC, we performed a GSEA analysis. According to the GO gene set, the high-risk group exhibited enrichment in complement activation, immunoglobulin complex, and immune response mediated by circulating immunoglobulin (**Figure 12A**), whereas the low-risk group showed enrichment in mitochondrial protein-containing complex and mitochondrial translation (**Figure 12B**). Considering the strong association between CLEC11A-derived immune signature and the immune microenvironment, we employed the ESTIMATE algorithm to evaluate the immune infiltration status in GC samples. As expected, CLEC11A-derived immune signature exhibited correlations with multiple immune microenvironment scores. In the high-risk group, the immune score, stromal score, and ESTIMATE score were significantly higher than the low-risk group (**Figure 12C**). Moreover, employing the ssGSEA algorithm, we observed that the high-risk group demonstrated significantly enhanced activities in pathways associated with T cell co-inhibition pathways, cytolytic activity, and inflammation promotion (**Figure 12D**). To further investigate the variations of immune cell infiltration between risk score subgroups, we quantified the abundance of infiltrating immune cells by CIBERSORT algorithm. As shown in **Figure 12E**, T cells follicular helper, T cells CD4 memory, and Macrophages M2 were more abundant in the high-risk group. Furthermore, our findings indicated a strong correlation between the signature genes and tumor-infilrating immune cells. Interestingly, CD86, CSF1R, TGFBR1, and TNFSF18 showed a positive correlation with macrophage M2, as depicted in **Figure 12F**.

### 3.13 Analysis of immunotherapy response and drug sensitivity

**Figure 13:**
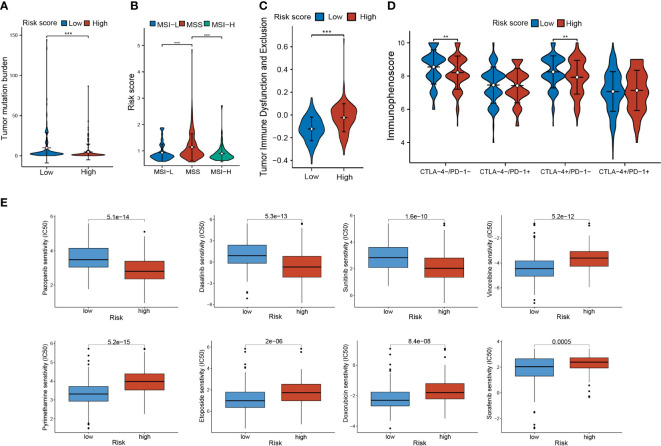
Immunotherapy response and drug sensitivity. **(A)** TMB in risk score subgroups. **(B)** The differences of risk scores in MSI-H/MSS/MSI-L. **(C)** The TIDE scores and **(D)** IPS in risk score subgroups. **(E)** Drug sensitivities of Pazopanib, Dasatinib, Sunitinib, Vinorelbine, Sorafenib, Doxorubicin, Pyrimethamine, and Etoposide in risk score subgroups. ** P < 0.01, *** P < 0.001.

To understand the response of CLEC11A-derived immune signature to immunotherapy, we assessed the relationships between risk score and TMB, MSI/MSS, TIDE, and IPS. The results revealed that the high-risk group demonstrated a lower TMB, a well-established predictor of immunotherapy response (**Figure 13A**). Among the three microsatellite types, the risk score exhibited the highest distribution in the MSS subgroup (**Figure 13B**). Moreover, high-risk scores exhibited higher TIDE scores, which indicated the reduced efficacy of immunotherapy (**Figure 13C**). Conducting IPS analysis, we discovered that the IPS in the low-risk group was elevated for CTLA4+/PD1-treatment, which indicated that patients in the low-risk group had a better response to anti-CTLA4 therapy compared to those in the high-risk group (**Figure 13D**).

Furthermore, we investigated the response of risk score subgroups to drug treatments, with a focus on currently clinically used medications. As shown in **Figure 13E**, we found that the patients in the low-risk score group had higher IC50 values for Pazopanib, Dasatinib, and Sunitinib, while patients in the high-risk score group had higher IC50 values for Vinorelbine, Sorafenib, Doxorubicin, Pyrimethamine, and Etoposide.

## 4 Discussion

This study presented the evidence that CLEC11A was up-regulated in GC tissues and cell lines. Our analysis also proved that CLEC11A expression indicated undesirable clinical outcomes in GC patients. These findings suggested the clinical significance of CLEC11A as a potential biomarker for GC prognosis.

During the progression of malignant tumor, dysregulated growth factor signaling drives uncontrolled growth and division of cancer cells (76). It is worth noting that the specific role of CLEC11A, acting as a stem cell growth factor in GC, remains largely unknown. Our data indicated that CLEC11A was involved in the genomic instability and epigenetic modification, which is closely associated with the progression of GC. Furthermore, to understand the biofunction of CLEC11A, we employed functional enrichment analysis and uncovered that the biological processes of CLEC11A co-expression genes were mainly concentrated in the extracellular matrix organization and primarily involved in the cancer signaling pathway. To confirm these findings, we knocked down the expression level of CLEC11A and uncovered the contributions of CLEC11A in promoting cell cycle, migration and invasion. In animal models, we observed that CLEC11A promoted the growth of subcutaneous tumors in mice. Collectively, these results strongly demonstrated the oncogenic role of CLEC11A in GC development.

TME refers to an intricate biological environment that surrounds the growing cancer cells, which comprises extracellular matrix, stromal cells, immune cells, blood vessels, and lymphatic networks (77, 78). It has been found that TME favours the proliferation and expansion of cancer cells (79). To investigate whether CLEC11A exerts its carcinogenic effect by affecting the TME of GC, we performed the ESTIMATE algorithm and found that the cluster with higher CLEC11A expression had a significantly higher ESTIMATE score. Results from TISIDB further revealed that in the microenvironment of GC, CLEC11A was negatively correlated with CD4+ T cells and positively correlated with macrophages, MDSCs, and Tregs. These findings were confirmed by flow cytometry, where the knock-down of CLEC11A led to an increase in intratumoral CD8+ and CD4+ T cells and a decrease in immunosuppressive cells (M2 macrophages, MDSCs, and Tregs). As is well known, T cells play a central role in the immune response, and a decrease in T lymphocytes in the TME allows tumor cells to escape immune system attack (80). Moreover, as immune suppressive cell populations, M2 macrophages, MDSCs, and Tregs can accumulate in the GC microenvironment, promoting tumor escape by blocking cytotoxic T cells attack against the tumor (12). Knockdown of CLEC11A reshaped the composition of immunocytes in the TME, indicating the potential impact of CLEC11A on immune responses. Thus, we investigated the impact of CLEC11A on immunotherapy for GC. Unsurprisingly, heightened expression of CLEC11A correlated with elevated Dysfunction and Exclusion scores in the TIDE algorithm, signifying increased immune system exclusion by the tumor and a diminished response to immunotherapy. The results from IPS scoring analyses revealed that GC patients with lower levels of CLEC11A were more likely to benefit from immunotherapy in two specific groups (CTLA4-/PD-1- and CTLA4+/PD-1-). Additionally, there existed a negative correlation between CLEC11A and TMB/MSI, both serving as markers that are associated with cancer neoantigens and predicting immune response (81, 82). These results presented the influence of CLEC11A on immunotherapy, suggesting its potential as a target for enhancing immune therapy.

Due to the heterogeneity of GC, the survival durations among patients exhibit huge distinction, which covers a range of 5 months to 10 years (83, 84). Patients suffering from early-stage localized GC have a 5-year overall survival rate of above 60%, while for those diagnosed with distant metastasis, it is fewer than 5% (85). Such substantial variations in survival durations lead to a great challenge for clinicians in predicting the prognosis of GC patients. Encouragingly, the exploration of reliable biomarkers through bioinformatics has demonstrated remarkable potential in clinical applications. For example, a tumor immunophenotyping-derived signature constructed by Wang et al. can effectively evaluate the prognosis and response of GC patients to neoadjuvant ICI therapy (86). Additionally, Sui et al. developed a prognosis signature associated with immunocytes based on the cachexia-related genes, providing a deeper understanding of the immune mechanisms underlying cachexia in GC (87). In the present study, based on the CLEC11A-derived immune genes, we identified a stable and robust 6-gene prognosis signature for GC patients by incorporating TCGA data and verified its practicability using the GEO datasets. The predictive performance from multiple GC cohorts suggest that our developed prognosis signature holds potential in dealing with the heterogeneous survival prognoses of GC. To further offer a quantitative method for predicting the prognosis of GC patients in clinical practice, we constructed a nomogram that combined CLEC11A-derived immune signature and clinical features, which accurately predicted the survival rate of GC patients.

Some biomarkers included in the signature (CD86, CSF1R, CXCR4, TGFBR1, and TGFB1) have been discovered to be correlated with GC. As early as 1998, Japanese scholars discovered that CD86 was highly expressed in various gastric cancer cell lines (88). Later, Yang et al. found that CD86 expression could induce tumor angiogenesis in GC by activating VEGF-A expression (89). As the receptor of colony-stimulating factor-1 (CSF1), CSF1R is associated with the occurrence and prognosis of GC (90). CSF1R can promote the proliferation, migration, and resistance to anoikis in GC cell lines (91). CXCR4, as a chemokine receptor, can bind to CXCL12 and result in increased invasiveness of GC (92–94). Furthermore, the cross-talk between CXCR4 and EGFR, as well as the downstream Akt/ERK signaling pathway, can also promote the migration of GC (95). The transforming growth factor-beta (TGF-β) signaling pathway plays a crucial role in cell cycle regulation, growth, differentiation, extracellular matrix synthesis, and immune response (96). As two members of this signaling pathway, TGFB1 and TGFBR1 are expressed at high levels in GC and are associated with the initiation, progression, and metastasis of GC (97–99). Through the TGF-β1 signaling pathway, GC may gain strength by inducing Tregs under hypoxic conditions, allowing tumor cells to escape immunosurveillance (100). In this study, based on these key genes, we calculated the risk score, which was discovered to be strongly associated with the TME score. Moreover, in accordance with the CIBERSORT algorithm, the high-risk group had an elevated abundance of M2 macrophage fraction and signature genes were positively correlated various immunesupressive cells. Hence, we demonstrated that the effect of signature genes on the poor survival of GC patients was probably related to the mediation in the tumor microenvironment. Collectively, these findings offer novel insights for further molecular biology research on the mechanisms through which the signature genes modulate the immune microenvironment of GC patients.

Based on the relationships between the risk score and TMB, MSI, we concluded that individuals with high-risk scores are less likely to benefit from immunotherapy. The TIDE score analysis also displayed similar results. Given this, we attempted to use gene expression data from GC to further investigate the candidate agents for GC patients with unfavorable prognoses. The findings indicated that GC patients in the high-risk group exhibited sensitivity to Pazopanib, while those in the low-risk group exhibited sensitivity to sorafenib. Currently, Pazopanib and Sorafenib are targeted agents utilized in the clinical management of GC. As tyrosine kinase inhibitors, both Pazopanib and Sorafenib can inhibit the vascular endothelial growth factor receptor (VEGFR), thereby inhibiting angiogenesis in GC (101, 102). The commonly used clinical parameters, grading, and staging systems that currently guide the treatment decisions for GC have certain limitations. Specifically, patients with the same cancer staging often exhibit significant differences in response to the same treatment (103). By combining our prognostic features, clinicians can go beyond existing staging systems and provide more accurate treatment for patients with GC.

## 5 Conclusion

In the present research, we explored the molecular features, oncogenic effects, and TME characteristics of CLEC11A in GC. Moreover, we established a CLEC11A-derived immune signature that exhibited accurate prognosis prediction in GC patients. Overall, our study uncovered the prognostic and immunological value of CLEC11A and provided a potential option to predict the clinical outcome of GC patients.

## Data availability statement

The datasets presented in this study can be found in online repositories. The names of the repository/repositories and accession number(s) can be found in the article/**Supplementary Material**.

## Ethics statement

The animal study was approved by the Animal Ethics Committee of Shantou University Medical College. The study was conducted in accordance with the local legislation and institutional requirements.

## Author contributions

QZ: Writing – original draft. ZG: Investigation, Writing – original draft. BL: Investigation, Writing – original draft. RC: Validation, Writing – original draft. WL: Validation, Writing – original draft. CH: Writing – review & editing. HW: Conceptualization, Funding acquisition, Project administration, Writing – review & editing.

## Supplementary material

Supplementary Figure 1 The correlation between the abundance of immune cells and the overall survival of GC patients in TCGA-STAD.
