# Supplementary figures and images for: Identification and characterization of CLEC11A and its derived immune signature in gastric cancer
Source: Front Immunol. 2024 Jan 29;15:1324959. doi: 10.3389/fimmu.2024.1324959 (PMC10859539; doi:10.3389/fimmu.2024.1324959)

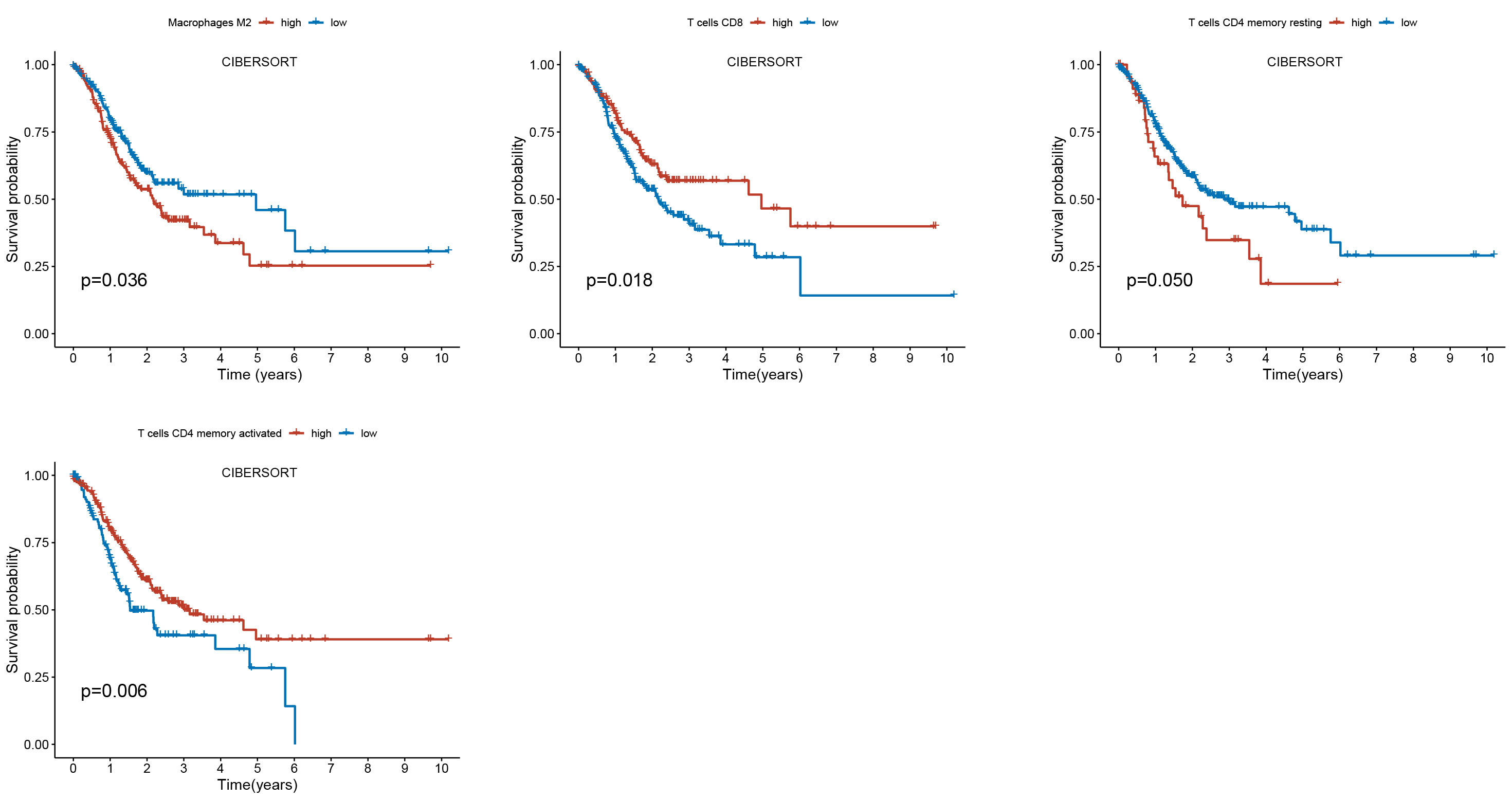

Supplement: Supplementary Figure 1 — The correlation between the abundance of immune cells and the overall survival of GC patients in TCGA-STAD. [file Image_1.jpeg]
